# Association of Hypertension with All-Cause Mortality among Hospitalized Patients with COVID-19

**DOI:** 10.3390/jcm9103136

**Published:** 2020-09-28

**Authors:** Enrique Rodilla, Alberto Saura, Iratxe Jiménez, Andrea Mendizábal, Araceli Pineda-Cantero, Elizabeth Lorenzo-Hernández, Maria del Pilar Fidalgo-Montero, Joaquín Fernandez López-Cuervo, Ricardo Gil-Sánchez, Elisa Rabadán-Pejenaute, Lucy Abella-Vázquez, Vicente Giner-Galvañ, Marta Nataya Solís-Marquínez, Ramon Boixeda, Andrés de la Peña-Fernández, Francisco Javier Carrasco-Sánchez, Julio González-Moraleja, José David Torres-Peña, María Esther Guisado-Espartero, Joaquín Escobar-Sevilla, Marcos Guzmán-García, María Dolores Martín-Escalante, Ángel Luis Martínez-González, José Manuel Casas-Rojo, Ricardo Gómez-Huelgas

**Affiliations:** 1 Internal Medicine Department, University Hospital of Sagunto, Universidad Cardenal Herrera-CEU, CEU Universities, 46520 Sagunto, Spain; albertosaura@hotmail.com (A.S.); iratxej@hotmail.com (I.J.); andreamendi@hotmail.com (A.M.); 2 Internal Medicine Department, Regional University Hospital of Málaga, 29010 Málaga, Spain; arapineda9@gmail.com (A.P.-C.); eli.lorenzo.hernandez@gmail.com (E.L.-H.); ricardogomezhuelgas@hotmail.com (R.G.-H.); 3 Internal Medicine Department, Henares Hospital, 28822 Coslada (Madrid), Spain; garrotefidalgo@telefonica.net; 4 Internal Medicine Department, Torrevieja University Hospital, 03186 Torrevieja (Alicante), Spain; joaquinfernandezcuervo11@gmail.com; 5 Internal Medicine Department, La Fe University Hospital, 46026 Valencia, Spain; rigilsan@gmail.com; 6 Internal Medicine Department, San Pedro Hospital, 26006 Logroño (La Rioja), Spain; elisapeje@gmail.com; 7 Internal Medicine Department, Ntra Sra Candelaria University Hospital, 38010 Santa Cruz de Tenerife, Spain; abellavazquez@gmail.com; 8 Internal Medicine Department, Hypertension and Cardiometabolic Risk Unit, San Juan de Alicante University Hospital, Miguel Hernández University, 03550 San Juan de Alicante (Alicante), Spain; ginervicgal@gmail.com; 9 Internal Medicine Department, San Agustin University Hospital, 33410 Avilés (Asturias), Spain; natayasolis@gmail.com; 10 Internal Medicine Department, Mataró Hospital, 08304 Mataró, Barcelona, Spain; rboixeda@ub.edu; 11 Internal Medicine Department, Son Llàtzer University Hospital, 07198 Palma de Mallorca, Spain; adelapen@hsll.es; 12 Internal Medicine Department, Juan Ramón Jiménez Hospital, 21005 Huelva, Spain; fjcarrascos@icloud.com; 13 Internal Medicine Department, Virgen de la Salud Hospital, 45004 Toledo, Spain; juliogmoraleja@gmail.com; 14 Internal Medicine Department, Lipids and Atherosclerosis Unit, Maimonides Biomedical Research Institute of Cordoba (IMIBIC), Reina Sofia University Hospital, University of Cordoba, Spain, CIBER Fisiopatología de la Obesidad y Nutrición (CIBEROBN), Instituto de Salud Carlos III, 14004 Cordoba, Spain; azarel_00@hotmail.com; 15 Internal Medicine Department, Infanta Margarita Hospital, 14940 Cabra (Córdoba), Spain; guesm53@hotmail.com; 16 Internal Medicine Department, Virgen de las Nieves University Hospital, 18014 Granada, Spain; escobarsevillaj@gmail.com; 17 Internal Medicine Department, San Juan de la Cruz Hospital, 23400 Úbeda (Jaén), Spain; m_guzman00@hotmail.es; 18 Internal Medicine Department, Costa del Sol Hospital, 29603 Marbella (Málaga), Spain; mmartinescalante@gmail.com; 19 Internal Medicine Department, León University Hospital Complex, 24008 León, Spain; garufa125@gmail.com; 20 Internal Medicine Department, Infanta Cristina University Hospital, 28981 Parla (Madrid), Spain; jm.casas@gmail.com

**Keywords:** hypertension, COVID-19, angiotensin-converting enzyme inhibitors (ACEIs), angiotensin II receptor blockers (ARBs), all-cause mortality

## Abstract

It is unclear to which extent the higher mortality associated with hypertension in the coronavirus disease (COVID-19) is due to its increased prevalence among older patients or to specific mechanisms. Cross-sectional, observational, retrospective multicenter study, analyzing 12226 patients who required hospital admission in 150 Spanish centers included in the nationwide SEMI-COVID-19 Network. We compared the clinical characteristics of survivors versus non-survivors. The mean age of the study population was 67.5 ± 16.1 years, 42.6% were women. Overall, 2630 (21.5%) subjects died. The most common comorbidity was hypertension (50.9%) followed by diabetes (19.1%), and atrial fibrillation (11.2%). Multivariate analysis showed that after adjusting for gender (males, OR: 1.5, *p* = 0.0001), age tertiles (second and third tertiles, OR: 2.0 and 4.7, *p* = 0.0001), and Charlson Comorbidity Index scores (second and third tertiles, OR: 4.7 and 8.1, *p* = 0.0001), hypertension was significantly predictive of all-cause mortality when this comorbidity was treated with angiotensin-converting enzyme inhibitors (ACEIs) (OR: 1.6, *p* = 0.002) or other than renin-angiotensin-aldosterone blockers (OR: 1.3, *p* = 0.001) or angiotensin II receptor blockers (ARBs) (OR: 1.2, *p* = 0.035). The preexisting condition of hypertension had an independent prognostic value for all-cause mortality in patients with COVID-19 who required hospitalization. ARBs showed a lower risk of lethality in hypertensive patients than other antihypertensive drugs.

## 1. Introduction

The novel coronavirus disease (COVID-19) is caused by severe acute respiratory syndrome coronavirus 2 (SARS-CoV-2). Worldwide, as of 14 August 2020, nearly 21 million (20,960,424) people had been diagnosed with COVID-19 and 760,371 had died [1].

One characteristic of this recent COVID-19 epidemic, described in the first reports coming out of China and Italy, was the observation that older patients with cardiovascular diseases (CVD) seemed to be highly represented, suggesting that there was a higher risk for worse outcomes of COVID-19 in this population [2,3]. Accordingly, hypertension (HT), which represents the single most important risk factor for CVD [4], has been repeatedly proposed as an independent prognostic factor of severe COVID-19 and has been included in clinical risk scores to predict the occurrence of critical illness in hospitalized patients with COVID-19 [5].

Several plausible arguments support the hypothesis of a causal association between HT and COVID-19. First, microvascular inflammation plays an important role in both the pathogenesis of HT and COVID-19, as illustrated by the high cytokine levels found in both HT and COVID-19 [6,7]. Second, angiotensin-converting enzyme 2 (ACE2) plays a pivotal role as a binding receptor for the cellular penetration of SARS-CoV-2 [8]. It is widely distributed on the respiratory epithelium as well as in the heart, kidney, and blood vessels [9]. Angiotensin-converting enzyme inhibitors (ACEIs) and angiotensin II receptor blockers (ARBs)—the most frequent antihypertensive drugs used in HT treatment [10]—have been linked in both animal models and in humans with up-regulation of ACE2 [11], thereby enhancing the ability of SARS-CoV-2 to infect cells and reducing the physiological degradation of angiotensin II. It has been claimed that the activation of this ACE/angiotensin/angiotensin II type-1 receptor (AT1R) axis [12] not only enhances susceptibility to but also the severity of SARS-CoV-2 infection [13].

The alternative hypothesis postulates that CVD, including HT, are simply confounding factors for the genuine association between older age and COVID-19 [14,15]. More evidence is still needed to support the idea that the association between HT and other CVD with COVID-19 is fully independent of age. In fact, prevalence of HT increases with advancing age, reaching a prevalence of >60% in people aged >60 years [16]. It is also well-known that age is a powerful risk factor for HT and other CVD. Furthermore, the causal role of age in explaining coronary heart disease and stroke increases in parallel with age [17].

Treatment of HT with renin-angiotensin-aldosterone system (RAAS) inhibitors might have a beneficial effect on COVID-19 patients. According to the ACE2/angiotensin 1–7/mas receptor axis theory, ACEIs and ARBs would contribute to counteracting the pro-inflammatory role of elevated angiotensin II levels as a result of decreased ACE2 activity [18]. The extent to which ACEIs and ARBs might have similar or different mechanisms of actions in COVID-19 patients is not known, but some authors predict a beneficial effect of ARBs compared to ACEIs [19], as angiotensin II represents the final product of the RAAS, whose pro-inflammatory effects should be avoided. Therefore, many scientific societies have published recommendations to continue antihypertensive treatment with ACEIs and ARBs in COVID-19 patients [20,21]. Interventional studies are now underway to test the anticipated benefit of adding ACEIs/ARBs to COVID-19 treatment, even in normotensive patients. 

The COVID-19 pandemic has hit Spain with unexpected severity; the country ranks fifth on the list of deaths per million inhabitants. The SEMI-COVID-19 Network was created by the Spanish Society of Internal Medicine (SEMI) to establish a nationwide, observational registry of patents who have been diagnosed with COVID-19. It includes epidemiological, laboratory, treatment, and outcome data [22]. 

The main objective of this study is to analyze whether HT represents an independent risk factor for death as a hard endpoint in patients hospitalized with SARS-CoV-2 in Spain. More specifically, it seeks to examine the effect previous treatment with ACEIs/ARBs may have on these patients. Additionally, the association between HT and ACEIs/ARBs with intensive care unit (ICU) admission and/or assisted ventilation was analyzed. 

## 2. Subjects and Methods

The SEMI-COVID-19 Registry is an ongoing nationwide, multicenter, observational, retrospective cohort registry. Information on the registry and data collection have been described elsewhere [22]. In summary, a total of 150 hospitals from the 17 regions that comprise Spain participated in the registry, thus assuring a representative sample of the entirety of the country. Inclusion criteria were age ≥18 years and first admission to a hospital in Spain with diagnosis of COVID-19 confirmed microbiologically by reverse transcription polymerase chain reaction (RT-PCR) testing of a nasopharyngeal sample, as per the recommendations of the World Health Organization [23]. The exclusion criteria included subsequent admissions of the same patient and denial or withdrawal of informed consent. Admission and treatment of patients took place at the discretion of the attending physicians based on their clinical judgment, local protocols, and the updated recommendations of the Spanish Ministry of Health. A total of 13121 consecutive patients were recruited from 1 March 2020 to 24 June 2020, when the last patient entered this study. Patients were 18 to 106 years of age.

The processing of personal data strictly complied with Spanish Law 14/2007, of July 3, on Biomedical Research; Regulation (EU) 2016/679 of the European Parliament and of the Council of 27 April 2016 on the protection of natural persons with regard to the processing of personal data and on the free movement of such data, and repealing Directive 95/46/EC (General Data Protection Regulation); and Spanish Organic Law 3/2018, of 5 December, on the Protection of Personal Data and the Guarantee of Digital Rights. The SEMI-COVID-19 Registry has been approved by the Provincial Research Ethics Committee of Málaga (Spain), following the recommendation of the Spanish Agency of Medicines and Medical Products (AEMPS, for its initials in Spanish). All patients gave their informed consent. When there were biosafety concerns and/or when the patient had already been discharged, verbal informed consent was requested and noted on the medical record. The conduct and reporting of the study were performed according to the STROBE statement guidelines. 

An online electronic data capture system (DCS) was developed on behalf of SEMI. After receiving training, at least one physician belonging to each hospital’s internal medicine department was responsible for acquiring and inputting the requested medical information into the DCS. This work was performed on a voluntary basis without remuneration. In order to ensure the highest possible quality of data collection, a database manager (JMCR) was designated and data verification procedures were implemented. The study’s scientific steering committee and an independent external agency performed database monitoring. Data analysis and logistics coordination were also carried out by independent external agencies. Alphanumeric sequences of characters based on identification codes were used to pseudoanonymize dissociated patient identifiable data so that the DCS did not contain any direct identifiers. A secure server hosts the database platform and all information is fully encrypted through a valid Transport Layer Security (TLS) certificate.

Approximately 300 variables were retrospectively collected under various headings: (1) inclusion criteria; (2) epidemiological data; (3) RT-PCR and serology data; (4) personal medical and medication history, including antihypertensive treatment categorized as ACEIs, ARBs, or other; (5) symptoms and physical examination findings at admission; (6) laboratory (blood gases, metabolic panel, complete blood count, and coagulation) and diagnostic imaging tests; (7) additional data at seven days after admission or at admission to the ICU; (8) pharmacological treatment and ventilator support during hospitalization; (9) complications during hospitalization; and (10) progress after discharge and/or 30 days from diagnosis. The raw and age-adjusted Charlson Comorbidity Index (CCI) score was calculated from the data collected [24]. A complete list of variables collected can be found in the source paper [22]. All-cause mortality during hospitalization versus hospital discharge was the primary endpoint. Secondary endpoints such as invasive or non-invasive ventilation and ICU admission were also explored. Time of follow-up was the period from admission to discharge or death. Mortality is expressed as the case fatality rate (CFR). The data this study is based on are available from the corresponding author upon reasonable request.

Continuous variables were tested for normal distribution using the Kolmogorov–Smirnov test. Results are shown as means (standard deviation, SD) or medians (25th to 75th percentile) for continuous variables and numbers (%) for categorical variables. 

To compare baseline demographic data and clinical characteristics among the different groups, we used analysis of variance (ANOVA) or the Kruskal–Wallis test for continuous variables. Differences in proportion were analyzed using the chi-square test. HT was categorized as absent or present; when present, it was further categorized into three groups according to treatment received: (a) non-ACEIs/ARBs, (b) ACEIs, and (c) ARBs. The association between these four categories (normotension, non-ACEIs/ARBs, ACEIs, and ARBs and death was analyzed using Kaplan–Meier survival curves; the log-rank test was calculated from baseline to time of death according to the HT groups. We used a multivariate logistic regression with all-cause mortality as the dependent variable to evaluate the role of normotension, previous treatment with non-ACEIs/ARBs, ACEIs, and ARBs and other comorbidities as predictor variables with a 95% confidence interval (CI). Variables with *p* < 0.1 on the univariate analysis were included. Sensitivity analysis was carried out through a second logistic regression with the composite secondary endpoint as dependent variable. All statistical analyses were performed using SPSS software (version 26.0, Chicago, IL, USA). A two-sided *p* value < 0.05 was considered statistically significant.

## 3. Results

### 3.1. Demographic Characteristics of the Study Population

**Figure 1:**
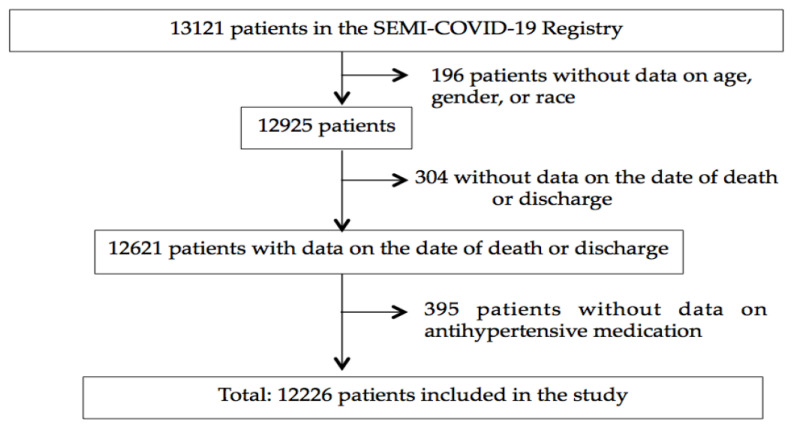
Patient inclusion flow chart.

The SEMI-COVID-19 Registry collected data from 13,121 records. Of these, 895 were missing information. In the end, 12,226 (93.2%) participants were included in the study. A subject inclusion flow chart can be seen in Figure 1. Demographic and baseline clinical features are listed in Table 1. Subjects’ mean age was 67.5 ± 16.1 years and 42.6% were women. The ethnicity of our study population was mostly white European origin (90.1%), followed by Latin American origin (8.2%). The data presented only represent patients who were hospitalized and discharged: 73.1% returned home, 5.9% continued their recovery in non-hospital healthcare institutions, and 21.0% died. Concerning information about terminal complications of COVID19 patients with fatal outcome, the most prevalent cause of death was adult respiratory distress syndrome (78.7%), followed at a considerable distance by acute renal failure (36.2%), multiorgan failure (27.0%), secondary bacterial pneumonia (22.1%), sepsis (20.5%), shock (16.2%), heart failure (HF, 14.8%), and cardiac arrhythmia (9.4%). Disseminated intravascular coagulation (3.3%), myocarditis (2.4%), acute coronary disease (2.3%), pulmonary embolism (1.8%), and stroke (1.7%) were also present, but far more rare. This proportion of deaths is in line with official data from the Spanish Ministry of Health as of 29 May 2020 (20,534 deaths out of 99,808 hospitalized patients, 20.6%) [25]. Mean time of hospitalization was 11.3 days (±10.3), ranging from 1 to 107 days.

The demographic and baseline clinical features of our population can be observed in Table 1. Among our subjects, HT was the most frequent comorbidity (50.9%), far ahead of diabetes mellitus (19.1%), atrial fibrillation (11.2%), chronic heart disease (CHD (8.0%), stroke (7.7%), HF (7.1%), chronic obstructive pulmonary disease (COPD) (7.0%), and chronic kidney disease (CKD) (6.0%). When stratifying our population by survival/non-survival, age was markedly higher in the non-survivor group. Likewise, all of the chronic comorbidities listed in Table 1 showed a significantly higher prevalence in the non-survivor group.

### 3.2. Outcomes

**Figure 2:**
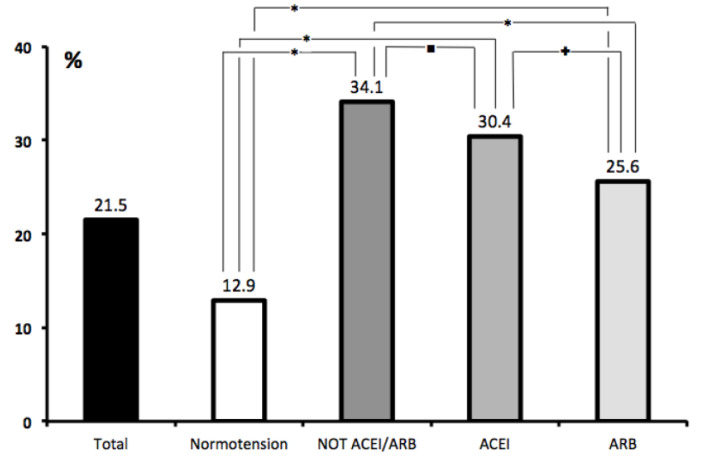
All-cause Mortality according to Hypertension status and previous antihypertensive treatment. * *p* < 0.0001; **+** *p* < 0.001; ⯀ *p* < 0.01.

To further characterize the association between HT and outcomes, we compared normotensive patients with hypertensive patients from all three hypertension categories. We observed a highly significant increase in the mortality rate of hypertensive versus normotensive subjects as well as worse outcomes in the non-ACEI/ARB group versus the ACEI and ARB groups. Interestingly, we also saw better outcomes among subjects in the ARB group compared to the ACEI group (Figure 2).

**Figure 3:**
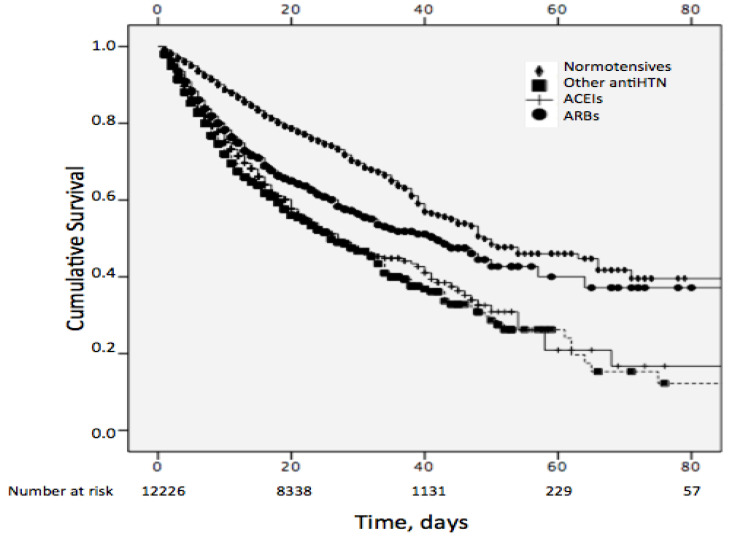
Kaplan–Meier curves in patients with/without hypertension according to previous antihypertensive treatment. Log rank *p* < 0.001.

Kaplan–Meier survival curves (Figure 3) according to blood pressure status confirm a clear increase in all-cause mortality in hypertensive patients in the non-ACEI/ARB group and the ACEI group compared to normotensive patients (log-rank *p* < 0001) from the very beginning of the observation period. Of note, the ARB group initially matches the curve of the former two groups, but there is a clear separation at around the third week of hospitalization, with figures then approaching the curve for normotensive patients. Remarkably, the maintenance of ARBs during the first two weeks and thereafter was very similar (47.0% vs. 44.3%, respectively).

Table 2 shows differences between normotensive and hypertensive patients according to the treatment groups. The figures indicate a similar distribution of comorbidities across the three treatment groups. When analyzing the ACEI and ARB groups, it is noteworthy that not only age, but also the Charlson Comorbidity Index scores were almost identical, pointing to a homogeneous distribution of comorbidities in both groups. There was a slightly higher percentage of males in the ACEI group (60.9% vs. 57.6%, *p* = 0.03).

Using all covariates with a significant association (*p* < 0.1) with all-cause mortality as the dependent variable, we performed a multivariate stepwise logistic regression analysis adjusting for age and gender (Table 3). We also included in–hospital use of ACEIs/ARBs to control for discontinuation of these drugs as confusion factors. In fact, only 44.9% of patients previously on ACEIs treatment and 46.4% of those on ARBs before hospitalization, maintained these antihypertensive drugs during the active phase of the disease. The two main factors that were independently predictive of death were the Charlson Comorbidity Index score and age. Male gender, atrial fibrillation, and HF remained significant determinants, but diabetes, COPD, and peripheral arterial disease did not. CKD was borderline significant. Of particular interest is the observation that compared to normotensive subjects, treated HT was significantly associated with increased all-cause mortality, independently of previous antihypertensive treatment. On the contrary, in-hospital treatment with ACEIs/ARBs exerted an independent and significant protective effect for fatal outcomes.

To increase the sensitivity of our study, we also analyzed a composite endpoint consisting of death, need for ventilatory support, and admission to the ICU. Table S1 (Supplementary Material) shows the results of the multivariate logistic regression for the composite endpoint outcome as the dependent variable. An analysis confirms the independent predictive value of age, Charlson Comorbidity Index score, sex, and antihypertensive treatment. On the other hand, HF, CKD, and atrial fibrillation were shown to have borderline significance with similar ORs. The independent protective effect of in-hospital use of ACEIs/ARBs was confirmed.

## 4. Discussion

To the best of our knowledge, this observational, cross-sectional, multicenter study constitutes the largest analysis of hospitalized, treated, and discharged COVID-19 patients worldwide. Data from 12,226 participants from 150 hospitals throughout the nation were collected, achieving a representative sample of the pandemic in Spain. Our analysis was strictly limited to patients who required in-hospital treatment. Focusing on the comorbidities associated with COVID-19 severity and using in-hospital all-cause mortality as a hard endpoint, the following conclusions can be drawn. 

First, a previous diagnosis of HT increased the risk of all-cause death in COVID-19 patients who required hospitalization on the order of approximately 20% and independently of age and other cardiovascular comorbidities, such as HF and atrial fibrillation. In line with the vast majority of studies, our results support the observation that COVID-19 patients are generally older and more fragile than the general population [2,26], as confirmed by the use of different scores reflecting concurrent chronic diseases [5]. In our study population, we observed that the higher the corrected Charlson Comorbidity Index score was, the higher all-cause mortality was. However, the specific association between HT and COVID-19 remains controversial. Some studies have linked presence of HT to worse outcomes in COVID-19 [27], whereas others consider HT to simply be a potential confounding factor for the real, causal relationship between age, cardiovascular disease, and increased mortality due to COVID-19 [15,28]. 

In a recent cross-sectional, observational, multicenter study, Iaccarino et al. [15] analyzed the comorbidities of 1591 COVID-19 patients in Italy. They found that HT was the most frequent preexisting condition (73.4%). Nevertheless, after adjusting for all other clinical conditions, only age, diabetes mellitus, chronic obstructive pulmonary disease, and chronic kidney disease—not HT—showed prognostic value for death from COVID-19. Although the work by Iaccarino et al. and our study share many similarities, such as the median age of survivors and non-survivors, the prevalence of baseline conditions, multivariate adjustment for all of them, and all-cause mortality as the primary endpoint, a fundamental difference was that up to 72.7% of the Italian study’s patients were still in the active phase of the COVID-19 disease. At that stage of the disease, the outcome should be at least considered uncertain in relation to a hard endpoint. In contrast, all of our patients had either been discharged or had died, therefore offering a definite result in terms of the principal outcome variable. Further arguments that could explain conflicting results between the studies include the fact that up to 7% of patients in the Italian study received outpatient treatment, as hospital admission was not an inclusion criterion. This could reflect a different degree of disease severity in those patients. 

Second, previous treatment with ACEIs/ARBs in hypertensive patients was not associated with a higher risk of all-cause mortality in hypertensive hospitalized COVID-19 patients compared to other antihypertensive drugs. Two recent papers addressing the relationship between previous treatment with ACEIs/ARBs and COVID-19 seem to confirm our observation; they found no increase in severity of COVID-19 in the group of patients treated with ACEIs/ARBs compared with other drugs for CVD [14,29]. It is important to underline that use of ACEIs/ARBs in both studies was not synonymous with HT, as the prevalence of HT in the group of patients treated with ACEIs/ARBs ranged between 58% and 71%. In other words, other diseases in which ACEIs/ARBs are also indicated as a baseline therapy were included in the analysis in these studies and may act as confounding factors of the benefit or harm of ACEIs/ARBs from the perspective of their role as treatment before hospital admission. In contrast, our study addressed the use of ACEIs/ARBs exclusively for HT and before admission. The results of both studies should therefore not be interpreted as arguments against the association between HT and COVID-19, but as evidence that previous treatment of CVD with ACEIs/ARBs does not relate to severity of or susceptibility to COVID-19 per se.

Third, the lowest risk of all-cause mortality in previously treated hypertensive COVID-19 patients was observed in the group of ARBs. Furthermore, patients previously treated with ARBs showed a tendency to become protective two weeks after admission. The fact that the so-called “cytokine storm” syndrome generally develops after the second week of COVID-19 infection [30], might explain the delay in the possible beneficial action of ARBs. Most studies include ACEIs/ARBs in a single group, as they share a common pathway. Nevertheless, angiotensin II has been shown to increase in COVID-19 and ARBs act on the final step of the RAAS system, precisely blocking the AT1-receptor for angiotensin II. It has therefore been claimed that for this reason, ARBs might be superior to ACEIs in improving COVID-19 prognosis [19].

As shown in this study, mortality increased in patients prevented from continuing their previous treatment with ACEIs/ARBs during their hospital stay. Therefore, a careful evaluation of medications used in hypertensive patients diagnosed with COVID 19 is mandatory. It is of capital importance to emphasize the scope of our study on HT treatment before hospital admission. In line with the evidence published to date, our study does not examine in-hospital management of COVID-19 patients as the main objective. Although the underlying mechanisms might be equally related to the ACE2/angiotensin 1-7/mas receptor axis, additional studies are necessary to evaluate confounding factors, especially the incidence of cardiovascular complications and in-hospital treatment with ACEIs/ARBs that may alter the extent of the association of HT and its previous treatment with all-cause mortality.

Our data about terminal complications contribute to understand the pathophysiological mechanisms of fatal outcomes in hospitalized COVID19 patients. Death was overwhelmingly caused by adult respiratory distress syndrome, suggesting that the cytokine storm may play a major role, although other pathways affecting especially the kidneys and the cardiovascular systems are also definitely involved.

The strengths of our study include the large number of participants, the use of a hard endpoint for analysis, and previous experience in handling databases by a scientific society. Nevertheless, its cross-sectional design, the high proportion of patients of white ethnicity, the unknown real spread of COVID-19 in outpatients, and the strict inclusion of COVID-19 patients requiring hospital admission do not allow for our results to be extrapolated to the general population. Further studies, using data from death certificates, should be carried out to further explore the association between hypertension and all-cause mortality at population level. Improving medical certification of cause of death across countries with an analysis protocol for uniform minimum data reporting is necessary for addressing the growing burden of the COVID19 pandemic [31].

## 5. Conclusions

Hypertension is associated with a higher risk for all-cause mortality independently of other comorbidities, sex, and age. Previous treatment with ACEIs/ARBs, compared to other antihypertensive drugs, does not alter outcomes in hypertensive patients. Compared to other antihypertensive drugs, hypertensive patients previously treated with angiotensin II receptor blockers (ARBs) had the lowest risk for all-cause mortality.

## Figures and Tables

**Table 1:** Demographic and baseline clinical features of the study population. COPD: chronic obstructive pulmonary disease; CKD: chronic kidney disease.

Variable	Total Study Population (*n* = 12226)	Non-Survivors (*n* = 2630)	Survivors (*n* = 9596)	*p* Value
Age, years	67.5 ± 16.1	79.6 ± 10.5	64.1 ± 15.7	0.0001
Women, %	42.6	38.1	43.8	0.0001
Hypertension, %	50.9	70.6	45.5	0.0001
Diabetes mellitus, %	19.1	28.2	16.6	0.0001
COPD, %	7.0	12.5	5.5	0.0001
CKD, %	6.0	12.5	4.3	0.0001
Coronary heart disease, %	8.0	14.1	6.3	0.0001
Heart failure, %	7.1	15.4	4.9	0.0001
Stroke, %	7.7	14.2	6.0	0.0001
Atrial fibrillation, %	11.2	21.7	8.3	0.0001
Peripheral vascular disease, %	4.7	9.0	3.5	0.0001
Charlson Comorbidity Index score	3.6 ± 2.7	5.7 ± 2.4	3.1 ± 2.5	0.0001

**Table 2:** Demographic and baseline clinical features of the study population. according to hypertension (HT) status and treatment. COPD: chronic obstructive pulmonary disease; CKD: chronic kidney disease; ACEI: Angiotensin-converting enzyme inhibitors; ARB: angiotensin II receptor blockers.

Variable	Normotensive Subjects (*n* = 6001)	Hypertensive Subjects			*p* Value
		Non-ACEI/ARB (*n* = 1987)	ACEI (*n* = 1983)	ARB (*n* = 2255)	
Death, %	12.9	34.1	30.4	25.6	0.0001
Age, years	60.1 ± 16.1	76.4 ± 12.7	73.5 ± 12.6	73.9 ± 11.8	0.0001
Women, %	41.8	48.5	39.1	42.4	0.0001
Hypertension, %	0	100	100	100	0.0001
Diabetes mellitus, %	9.3	25.5	29.3	30.4	0.0001
COPD, %	4.6	10.0	9.2	9.0	0.0001
CKD, %	1.8	12.5	7.3	10.5	0.0001
Coronary heart disease, %	3.1	13.0	12.9	12.1	0.0001
Heart failure, %	2.6	14.7	10.6	9.4	0.0001
Stroke, %	3.9	12.6	11.2	10.8	0.0001
Atrial fibrillation, %	4.9	21.4	15.1	15.3	0.0001
Peripheral vascular disease, %	2.2	7.1	6.5	7.5	0.0001
Charlson Comorbidity Index score	2.5 ± 2.3	5.1 ± 2.6	4.5 ± 2.5	4.6 ± 2.5	0.0001

**Table 3:** Association with in-hospital all-cause mortality. Univariate analysis and adjusted multivariate logistic regression model. CKD: chronic kidney disease; HF: heart failure.

Variable.	Univariate Analysis		Multivariate Analysis	
	OR (CI 95%)	*p* Value	OR (CI 95%)	*p* Value
Age, tertiles				
<60.9	1		1	
≥60.9 and <76.3	5.3 (4.38–6.30)	0.0001	2.0 (1.56–2.43)	0.0001
≥76.3	20.3 (17.08–24.16)	0.0001	4.7 (3.75–5.92)	0.0001
Blood pressure				
Normotension	1		1	
Non-ACEIs/ARBs	3.5 (3.11–3.95)	0.0001	1.3 (1.08–1.43)	0.002
ACEIs	3.0 (2.61–3.34)	0.0001	1.6 (1.35–1.85)	0.001
ARBs	2.3 (2.07–2.64)	0.0001	1.2 (1.01–1.38)	0.035
Charlson Comorbidity Index, tertiles				
<2	1		1	
≥2 and <5	11.2 (9.34–13.45)	0.0001	4.7 (3.73–5.86)	0.0001
≥5	28.2 (23.47–33.97)	0.0001	8.1 (6.37–10,36)	0.0001
Gender				
Female	1		1	
Male	1.3 (1.16–1.38)	0.0001	1.5 (1.39–1.71)	0.0001
ACEIs (in-hospital)				
no	1		1	
yes	1.1 (0.93–1.24)	0.357	0.6 (0.45–0.66)	0.0001
ARBs (in-hospital)				
no	1		1	
yes	0.86 (0.74–0.99)	0.046	0.5 (0.45–0.65)	0.0001
HF				
no	1		1	
yes	3.6 (3.08–4.09)	0.0001	1.2 (1.01–1.41)	0.037
Atrial fibrillation				
no	1		1	
yes	1.3 (1.16–1.38)	0.0001	1.2 (1.01–1.33)	0.034
CKD				
no	1		1	
yes	3.2 (2.75–3.73)	0.0001	1.2 (0.99–1.41)	0.068

## Supplementary Materials

The following are available online at https://www.mdpi.com/2077-0383/9/10/3136/s1, Table S1: Association with the composite endpoint. Univariate analysis and adjusted multivariate logistic regression model. CKD: chronic kidney disease; HF: heart failure.
