# Supplementary material for: Association of Hypertension with All-Cause Mortality among Hospitalized Patients with COVID-19
Source: J Clin Med. 2020 Sep 28;9(10):3136. doi: 10.3390/jcm9103136 (PMC7650567; doi:10.3390/jcm9103136)
Supplement: Supplementary file 1 [file jcm-09-03136-s001.zip › jcm-927890-supplementary.docx]

**Supplementary Materials**

**Table S1.** Association with the composite endpoint. Univariate analysis and adjusted multivariate logistic regression model. CKD: chronic kidney disease; HF: heart failure.

| **Variable** | **Univariate analysis** | | **Multivariate analysis** | |
| --- | --- | --- | --- | --- |
|  | **OR (CI95%)** | ***p* Value** | **OR (CI95%)** | ***p* Value** |
| Age, tertiles  < 60.9  ≥ 60.9 and < 76.3  ≥ 76.3 | 1  2.5 (2.21*–*2.82)  6.7 (5.94*–*7.50) | 0.0001  0.0001 | 1  1.5 (1.28*–*1.75)  2.9 (2.39*–*3.39) | 0.0001  0.0001 |
| Blood pressure  Normotension  Non-ACEIs/ARBs  ACEIs  ARBs | 1  2.7 (2.34*–*2.96)  2.2 (1.99*–*2.49)  1.9 (1.68*–*2.10) | 0.0001  0.0001  0.0001 | 1  1.3 (1.12*–*1.44)  1.4 (1.21*–*1.60)  1.2 (1.06*–*1.41) | 0.0001  0.0001  0.005 |
| Charlson Comorbidity Index, tertiles  < 2  ≥ 2 and < 5  ≥ 5 | 1  3.6 (3.24*–*4.05)  7.6 (6.77*–*8.60) | 0.0001  0.0001 | 1  2.0 (1.72*–*2.34)  3.1 (2.61*–*3.74) | 0.0001  0.0001 |
| Gender  Female  Male | 1  1.3 (1.23*–*1.44) | 0.0001 | 1  1.5 (1.39*–*1.67) | 0.0001 |

| ACEIs (in-hospital)  no  yes | 1  1.1 (0.97*–*1.27) | 0.128 | 1  0.7 (0.58*–*0.82) | 0.0001 |
| --- | --- | --- | --- | --- |
| ARBs (in-hospital)  no  yes | 1  0.83 (0.73*–*0.96) | 0.009 | 1  0.6 (0.48*–*0.67) | 0.0001 |

| HF  no  yes | 1  2.78 (2.42*–*3.20) | 0.0001 | 1  1.2 (1.00*–*1.39) | 0.047 |
| --- | --- | --- | --- | --- |
| Atrial fibrillation  no  yes | 1  2.4 (2.17*–*2.74) | 0.0001 | 1  1.1 (1.00*–*1.30) | 0.056 |

List of the SEMI-COVID-19 Network members

Coordinator of the SEMI-COVID-19 Registry: José Manuel Casas Rojo.

SEMI-COVID-19 Scientific Committee Members: José Manuel Casas Rojo, José Manuel Ramos Rincón, Carlos Lumbreras Bermejo, Jesús Millán Núñez-Cortés, Juan Miguel Antón Santos, and Ricardo Gómez Huelgas.

SEMI-COVID-19 Registry Coordinating Center: S & H Medical Science Service.

Members of the SEMI-COVID-19 Group

H. U. 12 de Octubre. Madrid

Paloma Agudo de Blas, Coral Arévalo Cañas, Blanca Ayuso, José Bascuñana Morejón, Samara Campos Escudero, María Carnevali Frías, Santiago Cossio Tejido, Borja de Miguel Campo, Carmen Díaz Pedroche, Raquel Diaz Simon, Ana García Reyne, Lucia Jorge Huerta, Antonio Lalueza Blanco, Jaime Laureiro Gonzalo, Carlos Lumbreras Bermejo, Guillermo Maestro de la Calle, Barbara Otero Perpiña, Diana Paredes Ruiz, Marcos Sánchez Fernández, and Javier Tejada Montes.

H. U. Gregorio Marañón. Madrid

Laura Abarca Casas, Álvaro Alejandre de Oña, Rubén Alonso Beato, Leyre Alonso Gonzalo, Jaime Alonso Muñoz, Crhistian Mario Amodeo Oblitas, Cristina Ausín García, Marta Bacete Cebrián, Jesús Baltasar Corral, Maria Barrientos Guerrero, Alejandro Bendala Estrada, María Calderón Moreno, Paula Carrascosa Fernández, Raquel Carrillo, Sabela Castañeda Pérez, Eva Cervilla Muñoz, Agustín Diego Chacón Moreno, Maria Carmen Cuenca Carvajal, Sergio de Santos, Andrés Enríquez Gómez, Eduardo Fernández Carracedo, María Mercedes Ferreiro-Mazón Jenaro, Francisco Galeano Valle, Alejandra Garcia, Irene Garcia Fernandez-Bravo, María Eugenia García Leoni, Maria Gomez Antunez, Candela González San Narciso, Anthony Alexander Gurjian, Lorena Jiménez Ibáñez, Cristina Lavilla Olleros, Cristina Llamazares Mendo, Sara Luis García, Víctor Mato Jimeno, Clara Millán Nohales, Jesús Millán Núñez-Cortés, Sergio Moragón Ledesma, Antonio Muiño Miguez, Cecilia Muñoz Delgado, Lucía Ordieres Ortega, Susana Pardo Sánchez, Alejandro Parra Virto, María Teresa Pérez Sanz, Blanca Pinilla Llorente, Sandra Piqueras Ruiz, Guillermo Soria Fernández-Llamazares, María Toledano Macías, Neera Toledo Samaniego, Ana Torres do Rego, Maria Victoria Villalba Garcia, Gracia Villarreal, and María Zurita Etayo.

Hospital Universitari de Bellvitge. L’Hospitalet de Llobregat

Xavier Corbella, Narcís Homs, Abelardo Montero, Jose María Mora-Luján, and Manuel Rubio Rivas.

H. U. La Paz-Cantoblanco-Carlos III. Madrid

Jorge Álvarez Troncoso, Francisco Arnalich Fernández, Francisco Blanco Quintana, Carmen Busca Arenzana, Sergio Carrasco Molina, Aranzazu Castellano Candalija, Germán Daroca Bengoa, Alejandro de Gea Grela, Alicia de Lorenzo Hernández, Alejandro Díez Vidal, Carmen Fernández Capitán, Maria Francisca García Iglesias, Borja González Muñoz, Carmen Rosario Herrero Gil, Juan María Herrero Martínez, Víctor Hontañón, Maria Jesús Jaras Hernández, Carlos Lahoz, Cristina Marcelo Calvo, Juan Carlos Martín Gutiérrez, Monica Martinez Prieto, Elena Martínez Robles, Araceli Menéndez Saldaña, Alberto Moreno Fernández, Jose Maria Mostaza Prieto, Ana Noblejas Mozo, Carlos Manuel Oñoro López, Esmeralda Palmier Peláez, Marina Palomar Pampyn, Maria Angustias Quesada Simón, Juan Carlos Ramos Ramos, Luis Ramos Ruperto, Aquilino Sánchez Purificación, Teresa Sancho Bueso, Raquel Sorriguieta Torre, Clara Itziar Soto Abanedes, Yeray Untoria Tabares, Marta Varas Mayoral, and Julia Vásquez Manau.

C. H. U. de Albacete. Albacete

Jose Luis Beato Pérez and Maria Lourdes Sáez Méndez.

Complejo Asistencial de Segovia. Segovia

Eva María Ferreira Pasos, Daniel Monge Monge, and Alba Varela García.

H. U. Puerta de Hierro. Majadahonda

María Álvarez Bello, Ane Andrés Eisenhofer, Ana Arias Milla, Isolina Baños Pérez, Javier Bilbao Garay, Silvia Blanco Alonso, Jorge Calderón Parra, Alejandro Callejas Díaz, José María Camino Salvador, Mª Cruz Carreño Hernández, Valentín Cuervas-Mons Martínez, Sara de la Fuente Moral, Miguel del Pino Jimenez, Alberto Díaz de Santiago, Itziar Diego Yagüe, Ignacio Donate Velasco, Ana María Duca, Pedro Durán del Campo, Gabriela Escudero López, Esther Expósito Palomo, Ana Fernández Cruz, Esther Fiz Benito, Andrea Fraile López, Amy Galán Gómez, Sonia García Prieto, Claudia García Rodríguez-Maimón, Miguel Ángel García Viejo, Javier Gómez Irusta, Edith Vanessa Gutiérrez Abreu, Isabel Gutiérrez Martín, Ángela Gutiérrez Rojas, Andrea Gutiérrez Villanueva, Jesús Herráiz Jiménez, Pedro Laguna del Estal, Mª Carmen Máinez Sáiz, Cristina Martín Martín, María Martínez Urbistondo, Fernando Martínez Vera, Susana Mellor Pita, Patricia Mills Sánchez, Esther Montero Hernández, Alberto Mora Vargas, Cristina Moreno López, Alfonso Ángel-Moreno Maroto, Victor Moreno-Torres Concha, Ignacio Morrás De La Torre, Elena Múñez Rubio, Ana Muñoz Gómez, Rosa Muñoz de Benito, Alejandro Muñoz Serrano, Jose María Palau Fayós, Ilduara Pintos Pascual, Antonio Ramos Martínez, Isabel Redondo Cánovas del Castillo, Alberto Roldán Montaud, Lucía Romero Imaz, Yolanda Romero Pizarro, Mónica Sánchez Santiuste, David Sánchez Órtiz, Enrique Sánchez Chica, Patricia Serrano de la Fuente, Pablo Tutor de Ureta, Ángela Valencia Alijo, Mercedes Valentín-Pastrana Aguilar, Juan Antonio Vargas Núñez, Jose Manuel Vázquez Comendador, Gema Vázquez Contreras, and Carmen Vizoso Gálvez.

H. Miguel Servet. Zaragoza

Gonzalo Acebes Repiso, Uxua Asín Samper, María Aranzazu Caudevilla Martínez, José Miguel García Bruñén, Rosa García Fenoll, Jesús Javier González Igual, Laura Letona Giménez, Mónica Llorente Barrio, and Luis Sáez Comet.

H. U. La Princesa. Madrid

María Aguilera García, Ester Alonso Monge, Jesús Álvarez Rodríguez, Claudia Alvarez Varela, Miquel Berniz Gòdia, Marta Briega Molina, Marta Bustamante Vega, Jose Curbelo, Alicia de las Heras Moreno, Ignacio Descalzo Godoy, Alexia Constanza Espiño Alvarez, Ignacio Fernández Martín-Caro, Alejandra Franquet López-Mosteiro, Gonzalo Galvez Marquez, María J. García Blanco, Yaiza García del Álamo Hernández, Clara García-Rayo Encina, Noemí Gilabert González, Carolina Guillamo Rodríguez, Nicolás Labrador San Martín, Manuel Molina Báez, Carmen Muñoz Delgado, Pedro Parra Caballero, Javier Pérez Serrano, Laura Rabes Rodríguez, Pablo Rodríguez Cortés, Carlos Rodriguez Franco, Emilia Roy-Vallejo, Monica Rueda Vega, Aresio Sancha Lloret, Beatriz Sánchez Moreno, Marta Sanz Alba, Jorge Serrano Ballester, Alba Somovilla, Carmen Suarez Fernández, Macarena Vargas Tirado, and Almudena Villa Marti.

H. U. de A Coruña. A Coruña

Alicia Alonso Álvarez, Olaya Alonso Juarros, Ariadna Arévalo López, Carmen Casariego Castiñeira, Ana Cerezales Calviño, Marta Contreras Sánchez, Ramón Fernández Varela, Santiago J. Freire Castro, Ana Padín Trigo, Rafael Prieto Jarel, Fátima Raad Varea, Laura Ramos Alonso, Francisco Javier Sanmartín Pensado, and David Vieito Porto.

H. Clínico San Carlos. Madrid

Inés Armenteros Yeguas, Javier Azaña Gómez, Julia Barrado Cuchillo, Irene Burruezo López, Noemí Cabello Clotet, Alberto E. Calvo Elías, Elpidio Calvo Manuel, Carmen María Cano de Luque, Cynthia Chocron Benbunan, Laura Dans Vilan, Ester Emilia Dubon Peralta, Vicente Estrada Pérez, Santiago Fernandez-Castelao, Marcos Oliver Fragiel Saavedra, José Luis García Klepzig, Maria del Rosario Iguarán Bermúdez, Esther Jaén Ferrer, Rubén Ángel Martín Sánchez, Manuel Méndez Bailón, Maria José Nuñez Orantos, Carolina Olmos Mata, Eva Orviz García, David Oteo Mata, Cristina Outon González, Juncal Perez-Somarriba, Pablo Pérez Mateos, Maria Esther Ramos Muñoz, Xabier Rivas Regaira, Iñigo Sagastagoitia Fornie, Alejandro Salinas Botrán, Miguel Suárez Robles, Maddalena Elena Urbano, and Miguel Villar Martínez.

H. Infanta Sofía. S. S. de los Reyes

Rafael del Castillo Cantero, Rebeca Fuerte Martínez, Arturo Muñoz Blanco, José Francisco Pascual Pareja, Isabel Perales Fraile, Isabel Rábago Lorite, Llanos Soler Rangel, Inés Suárez García, and Jose Luis Valle López.

Hospital Universitario Dr. Peset. Valencia

Juan Alberto Aguilera Ayllón, Arturo Artero, María del Mar Carmona Martín, María José Fabiá Valls, Maria de Mar Fernández Garcés, Ana Belén Gómez Belda, Ian López Cruz, Manuel Madrazo López, Elisabet Mateo Sanchis, Jaume Micó Gandia, Laura Piles Roger, Adela Maria Pina Belmonte, and Alba Viana García.

Hospital Clínico de Santiago. Santiago de Compostela

Maria del Carmen Beceiro Abad, Maria Aurora Freire Romero, Sonia Molinos Castro, Emilio Manuel Paez Guillan, María Pazo Nuñez, and Paula Maria Pesqueira Fontan.

H. Nuestra Señora del Prado. Talavera de la Reina

Sonia Casallo Blanco and Jeffrey Oskar Magallanes Gamboa.

H. U. Ramón y Cajal. Madrid

Luis Fernando Abrego Vaca, Ana Andréu Arnanz, Octavio Arce García, Marta Bajo González, Pablo Borque Sanz, Alberto Cozar Llisto, Sonia de Pedro Baena, Beatriz Del Hoyo Cuenda, María Alejandra Gamboa Osorio, Isabel García Sánchez, Andrés González García, Oscar Alberto López Cisneros, Miguel Martínez Lacalzada, Borja Merino Ortiz, Jimena Rey-García, Elisa Riera González, Cristina Sánchez Díaz, Grisell Starita Fajardo, Cecilia Suárez Carantoña, Adrian Viteri Noel, and Svetlana Zhilina Zhilina.

Hospital Royo Villanova. Zaragoza

Nicolás Alcalá Rivera, Anxela Crestelo Vieitez, Esther del Corral, Jesús Díez Manglano, Isabel Fiteni Mera, Maria del Mar Garcia Andreu, Martin Gerico Aseguinolaza, Claudia Josa Laorden, Raul Martinez Murgui, and Marta Teresa Matía Sanz.

H. U. Infanta Cristina. Parla

Juan Miguel Antón Santos, Ana Belén Barbero Barrera, Coralia Bueno Muiño, Ruth Calderón Hernaiz, Irene Casado Lopez, José Manuel Casas Rojo, Andrés Cortés Troncoso, Mayte de Guzmán García-Monge, Francesco Deodati, Gonzalo García Casasola Sánchez, Elena Garcia Guijarro, Davide Luordo, María Mateos González, Jose A Melero Bermejo, Lorea Roteta García, Elena Sierra Gonzalo, and Javier Villanueva Martínez.

H. de Cabueñes. Gijón

Ana María Álvarez Suárez, Carlos Delgado Vergés, Rosa Fernandez-Madera Martínez, Eva Fonseca Aizpuru, Alejandro Gómez Carrasco, Cristina Helguera Amezua, Juan Francisco López Caleya, María del Mar Martínez López, Aleida Martínez Zapico, Carmen Olabuenaga Iscar, María Luisa Taboada Martínez, and Lara María Tamargo Chamorro.

Hospital de Urduliz Alfredo Espinosa. Urdúliz

María Aparicio López, Asier Aranguren Arostegui, Paula Arriola Martínez, Gorka Arroita Gonzalez, Mª Soledad Azcona Losada, Miriam García Gómez, Eduardo Garcia Lopez, Amalur Iza Jiménez, Alazne Lartategi Iraurgi, Esther Martinez Becerro, Itziar Oriñuela González, Isabel María Portales Fernández, Pablo Ramirez Sánchez, Beatriz Ruiz Estévez, and Cristian Vidal Núñez.

Hospital Regional Universitario de Málaga. Málaga

Mª Mar Ayala Gutiérrez, Rosa Bernal López, José Bueno Fonseca, Verónica Andrea Buonaiuto, Luis Francisco Caballero Martínez, Lidia Cobos Palacios, Clara Costo Muriel, Francis de Windt, Ana Teresa Fernandez-Truchaud Christophel, Paula García Ocaña, Ricardo Gómez Huelgas, Javier Gorospe García, Maria Dolores López Carmona, Pablo López Quirantes, Almudena López Sampalo, Elizabeth Lorenzo Hernández, Juan José Mancebo Sevilla, Jesica Martin Carmona, Luis Miguel Pérez-Belmonte, Araceli Pineda Cantero, Michele Ricci, and Jaime Sanz Cánovas

H. Santa Marina. Bilbao

Maria Areses Manrique, Ainara Coduras Erdozain, and Ane Elbire Labirua-Iturburu Ruiz.

H. Moisès Broggi. Sant Joan Despí

Judit Aranda Lobo, Jose Loureiro Amigo, Isabel Oriol Bermúdez, Melani Pestaña Fernández, Nicolas Rhyman, and Nuria Vázquez Piqueras.

Hospital HLA Moncloa. Madrid

Guillermo Estrada, Teresa Garcia Delange, Isabel Jimenez Martinez, Carmen Martinez Cilleros, and Nuria Parra Arribas.

H. del Henares. Coslada

Jesús Ballano Rodríguez-Solís, Luis Cabeza Osorio, María del Pilar Fidalgo Montero, Mª Isabel Fuentes Soriano, Erika Esperanza Lozano Rincon, Ana Martín Hermida, Jesus Martinez Carrilero, Jose Angel Pestaña Santiago, Manuel Sánchez Robledo, Patricia Sanz Rojas, Nahum Jacobo Torres Yebes, and Vanessa Vento.

H. U. Torrevieja. Torrevieja

Julio César Blázquez Encinar and Joaquín Fernández López-Cuervo.

H. U. La Fe. Valencia

Dafne Cabañero, María Calabuig Ballester, Pascual Císcar Fernández, Ricardo Gil Sánchez, Marta Jiménez Escrig, Cristina Marín Amela, Laura Parra Gómez, Carlos Puig Navarro, and José Antonio Todolí Parra.

H. San Pedro. Logroño

Diana Alegre González, Irene Ariño Pérez de Zabalza, Sergio Arnedo Hernández, Jorge Collado Sáenz, Beatriz Dendariena, Marta Gómez del Mazo, Iratxe Martínez de Narvajas Urra, Sara Martínez Hernández, Estela Menendez Fernández, Jose Luís Peña Somovilla, and Elisa Rabadán Pejenaute.

Hospital Universitario Ntra Sra Candelaria. Santa Cruz de Tenerife

Lucy Abella, Andrea Afonso Díaz, Selena Gala Aguilera Garcia, Marta Bethencourt Feria, Eduardo Mauricio Calderón Ledezma, Sara Castaño Perez, Guillermo Castro Gainett, José Manuel del Arco Delgado, Joaquín Delgado Casamayor, Diego Garcia Silvera, Alba Gómez Hidalgo, Marcelino Hayek Peraza, Carolina Hernández Carballo, Rubén Hernández Luis, Francisco Javier Herrera Herrera, Maria del Mar Lopez Gamez, Julia Marfil Daza, María José Monedero Prieto, María Blanca Monereo Muñoz, María de la Luz Padilla Salazar, Daniel Rodríguez Díaz, Alicia Tejera, and Laura Torres Hernández.

H. U. San Juan de Alicante. San Juan de Alicante

David Balaz, David Bonet Tur, Carles García Cervera, David Francisco García Núñez, Vicente Giner Galvañ, Angie Gómez Uranga, Javier Guzmán Martínez, Isidro Hernández Isasi, Lourdes Lajara Villar, Juan Manuel Núñez Cruz, Sergio Palacios Fernández, Juan Jirge Peris García, Andrea Riaño Pérez, José Miguel Seguí Ripoll, and Philip Wikman-Jorgensen.

H. U. San Agustin. Avilés

Andrea Álvarez García, Víctor Arenas García, Alba Barragán Mateos, Demelsa Blanco Suárez, María Caño Rubia, Jaime Casal Álvarez, David Castrodá Copa, José Ferreiro Celeiro, Natalia García Arenas, Raquel García Noriega, Joaquin Llorente García, Irene Maderuelo Riesco, Paula Martinez Garcia, Maria Jose Menendez Calderon, Diego Eduardo Olivo Aguilar, Marta Nataya Solís Marquínez, Luis Trapiella Martínez, Andrés Astur Treceño García, and Juan Valdés Bécares.

H. de Mataró. Mataró

Raquel Aranega González, Ramon Boixeda, Carlos Lopera Mármol, Marta Parra Navarro, Ainhoa Rex Guzmán, and Aleix Serrallonga Fustier.

H. U. Son Llàtzer. Palma de Mallorca

Andrés de la Peña Fernández and Almudena Hernández Milián.

H. Virgen de la Salud. Toledo

Ana Maria Alguacil Muñoz, Marta Blanco Fernández, Veronica Cano, Ricardo Crespo Moreno, Fernando Cuadra Garcia-Tenorio, Blanca Díaz-Tendero Nájera, Raquel Estévez González, María Paz García Butenegro, Alberto Gato Díez, Verónica Gómez Caverzaschi, Piedad María Gómez Pedraza, Julio González Moraleja, Raúl Hidalgo Carvajal, Patricia Jiménez Arandq, Raquel Labra González, Áxel Legua Caparachini, Pilar Lopez Castañeyra, Agustín Lozano Ancin, Jose Domingo Martin Garcia, Cristina Morata Romero, María Jesús Moya Saiz, Helena Moza Moríñigo, Gemma Muñiz Nicolás, Enriqueta Muñoz Platon, Filomena Oliveri, Elena Ortiz Ortiz, Raúl Perea Rafael, Pilar Redondo Galán, María Antonia Sepulveda Berrocal, Vicente Serrano Romero de Ávila, Pilar Toledano Sierra, Yamilex Urbano Aranda, Jesús Vázquez Clemente, and Carmen Yera Bergua.

H. Juan Ramón Jiménez. Huelva

Francisco Javier Bejarano Luque, Francisco Javier Carrasco-Sánchez, Mercedes de Sousa Baena, Jaime Díaz Leal, Aurora Espinar Rubio, Maria Franco Huertas, Juan Antonio García Bravo, Andrés Gonzalez Macías, Encarnación Gutiérrez Jiménez, Alicia Hidalgo Jiménez, Constantino Lozano Quintero, Carmen Mancilla Reguera, Francisco Javier Martínez Marcos, Francisco Muñoz Beamud, Maria Perez Aguilera, Alícia Perez Jiménez, Virginia Rodríguez Castaño, Alvaro Sánchez de Alcazar del Río, and Leire Toscano Ruiz.

H. U. Reina Sofía. Córdoba

Antonio Pablo Arenas de Larriva, Pilar Calero Espinal, Javier Delgado Lista, María Jesús Gómez Vázquez, Jose Jiménez Torres, Laura Martín Piedra, Javier Pascual Vinagre, María Elena Revelles Vílchez, Juan Luis Romero Cabrera, and José David Torres Peña.

Hospital Infanta Margarita. Cabra

María Esther Guisado Espartero, Lorena Montero Rivas, Maria de la Sierra Navas Alcántara, and Raimundo Tirado-Miranda.

H. U. Virgen de las Nieves. Granada

Pablo Conde Baena, Joaquin Escobar Sevilla, Laura Gallo Padilla, Patricia Gómez Ronquillo, Pablo González Bustos, María Navío Botías, Jessica Ramírez Taboada, and Mar Rivero Rodrígez.

Hospital Costa del Sol. Marbella

Victoria Augustín Bandera and María Dolores Martín Escalante.

H. San Juan de la Cruz. Úbeda

Marcos Guzmán Garcia and Francisco Javier Vicente Hernández.

Complejo Asistencial Universitario de León. León

Rosario Maria García Die, Manuel Martin Regidor, Angel Luis Martínez Gonzalez, Alberto Muela Molinero, Raquel Rodríguez Díez, and Beatriz Vicente Montes.

Hospital Clinic Barcelona. Barcelona

Júlia Calvo Jiménez, Aina Capdevila Reniu, Irene Carbonell De Boulle, Emmanuel Coloma Bazán, Joaquim Fernández Solà, Cristina Gabara Xancó, Joan Ribot Grabalosa, and Olga Rodríguez Núñez.

C. H. U. de Ferrol. Ferrol

Hortensia Alvarez Diaz, Tamara Dalama Lopez, Estefania Martul Pego, Carmen Mella Pérez, Ana Pazos Ferro, Sabela Sánchez Trigo, Dolores Suarez Sambade, Maria Trigas Ferrin, Maria del Carmen Vázquez Friol, and Laura Vilariño Maneiro.

Hospital Marina Baixa. Villajoyosa

Javier Ena and Jose Enrique Gómez Segado.

Hospital del Tajo. Aranjuez

Ruth Gonzalez Ferrer and Raquel Monsalvo Arroyo.

Hospital Insular de Gran Canaria. Las Palmas G. C.

Marina Aroza Espinar, Jorge Orihuela Martín, Carlos Jorge Ripper, and Selena Santana Jiménez.

H. U. Marqués de Valdecilla. Santander

Marta Fernández-Ayala Novo, José Javier Napal Lecumberri, Nuria Puente Ruiz, Jose Riancho, and Isabel Sampedro Garcia.

Hospital Torrecárdenas. Almería

Luis Felipe Díez García, Iris El Attar Acedo, Bárbara Hernandez Sierra, and Carmen Mar Sánchez Cano.

H. U. Severo Ochoa. Leganés

Yolanda Casillas Viera, Lucía Cayuela Rodríguez, Carmen de Juan Alvarez, Gema Flox Benitez, Laura García Escudero, Juan Martin Torres, Patricia Moreira Escriche, Susana Plaza Canteli, and M Carmen Romero Pérez.

Hospital Valle del Nalón. Riaño (Langreo)

Sara Fuente Cosío, César Manuel Gallo Álvaro, Julia Lobo García, and Antía Pérez Piñeiro.

H. U. del Vinalopó. Elche

Francisco Amorós Martínez, Erika Ascuña Vásquez, Jose Carlos Escribano Stablé, Adriana Hernández Belmonte, Ana Maestre Peiró, Raquel Martínez Goñi, M. Carmen Pacheco Castellanos, Bernardino Soldan Belda, and David Vicente Navarro.

Hospital Alto Guadalquivir. Andújar

Begoña Cortés Rodríguez.

H. Francesc de Borja. Gandia

Alba Camarena Molina, Simona Cioaia, Anna Ferrer Santolalia, José María Frutos Pérez, Eva Gil Tomás, Leyre Jorquer Vidal, Marina Llopis Sanchis, Mari Ángeles Martínez Pascual, Alvaro Navarro Batet, Mari Amparo Perea Ribis, Ricardo Peris Sanchez, José Manuel Querol Ribelles, Silvia Rodriguez Mercadal, and Ana Ventura Esteve.

H. G. U. de Castellón. Castellón de la Plana

Jorge Andrés Soler, Marián Bennasar Remolar, Alejandro Cardenal Álvarez, Daniela Díaz Carlotti, María José Esteve Gimeno, Sergio Fabra Juana, Paula García López, María Teresa Guinot Soler, Daniela Palomo de la Sota, Guillem Pascual Castellanos, Ignacio Pérez Catalán, Celia Roig Martí, Paula Rubert Monzó, Javier Ruiz Padilla, Nuria Tornador Gaya, and Jorge Usó Blasco.

H. Santa Bárbara. Soria

Marta Leon Tellez.

C. A. U. de Salamanca. Salamanca

Gloria María Alonso Claudio, Víctor Barreales Rodríguez, Cristina Carbonell Muñoz, Adela Carpio Pérez, María Victoria Coral Orbes, Daniel Encinas Sánchez, Sandra Inés Revuelta, Miguel Marcos Martín, José Ignacio Martín González, José Ángel Martín Oterino, Leticia Moralejo Alonso, Sonia Peña Balbuena, María Luisa Pérez García, Ana Ramon Prados, Beatriz Rodríguez-Alonso, Ángela Romero Alegría, Maria Sanchez Ledesma, and Rosa Juana Tejera Pérez.

H. U. de Canarias. Santa Cruz de Tenerife

Julio Cesar Alvisa Negrin, José Fernando Armas González, Lourdes González Navarrete, Iballa Jiménez, María Candelaria Martín González, Miguel Nicolas Navarrete Lorite, Paula Ortega Toledo, Onán Pérez Hernández, and Alina Pérez Ramírez.

C. H. U. de Badajoz. Badajoz

Rafael Aragon Lara, Inmaculada Cimadevilla Fernandez, Juan Carlos Cira García, Gema Maria García García, Julia Gonzalez Granados, Beatriz Guerrero Sánchez, Francisco Javier Monreal Periáñez, and Maria Josefa Pascual Perez.

H. U. del Sureste. Arganda del Rey

Jon Cabrejas Ugartondo, Ana Belén Mancebo Plaza, Arturo Noguerado Asensio, Bethania Pérez Alves, and Natalia Vicente López.

H. U. Quironsalud Madrid. Pozuelo de Alarcón (Madrid)

Pablo Guisado Vasco, Ana Roda Santacruz, and Ana Valverde Muñoz.

H. de Poniente. Almería

Juan Antonio Montes Romero, Encarna Sánchez Martín, Jose Luis Serrano Carrillo de Albornoz, and Manuel Jesus Soriano Pérez.

H. U. Lucus Augusti. Lugo

Raquel Gómez Méndez and Ana Rodríguez Álvarez.

H. San Pedro de Alcántara. Cáceres

Angela Agea Garcia, Javier Galán González, Luis Gámez Salazar, Eva Garcia Sardon, Antonio González Nieto, Itziar Montero Días, Selene Núñez Gaspar, and Alvaro Santaella Gomez.

H. de Pozoblanco. Pozoblanco

José Nicolás Alcalá Pedrajas, Antonia Márquez García, and Inés Vargas.

H. Virgen de los Lirios. Alcoy (Alicante)

Mª José Esteban Giner.

Hospital Doctor José Molina Orosa. Arrecife (Lanzarote)

Virginia Herrero García and Berta Román Bernal.

H. Nuestra Señora de Sonsoles. Ávila

Alaaeldeen Abdelhady Kishta.

H. G. U. de Elda. Elda

Carmen Cortés Saavedra, Jennifer Fernández Gómez, Borja González López, María Soledad Hernández Garrido, Ana Isabel López Amorós, Maria de los Reyes Pascual Pérez, and Andrea Torregrosa García.

H. U. Puerta del Mar. Cádiz

José Antonio Girón González, Susana Fabiola Pascual Perez, Cristina Rodríguez Fernández-Viagas, and Maria José Soto Cardenas.

H. Parc Tauli. Sabadell

Francisco Epelde and Isabel Torrente

Hospìtal de Montilla. Montilla

Ana Cristina Delgado Zamorano, Beatriz Gómez Marín, Adrián Montaño Martínez, and Jose Luis Zambrana García.

H. Infanta Elena. Huelva

María Gloria Rojano Rivero.

H. de la Axarquía. Vélez-Málaga

Antonio Lopez Ruiz.

H. Virgen del Mar. Madrid

Thamar Capel Astrua, Paola Tatiana Garcia Giraldo, Maria Jesus Gonzalez Juarez, Victoria Marquez Fernandez, and Ada Viviana Romero Echevarry.

Hospital do Salnes. Vilagarcía de Arousa

Vanesa Alende Castro, Ana María Baz Lomba, Ruth Brea Aparicio, Marta Fernandez Morales, Jesus Manuel Fernandez Villar, Maria Teresa Lopez Monteagudo, Cristina Pérez García, Lorena María Rodríguez Ferreira, Diana Sande Llovo, and Maria Begoña Valle Feijoo.

H. La Fuenfría. Cercedilla

Daniel Arregui Gallego, Jorge Blanco Briones, Gonzalo M Muzquiz Rueda, Isabel Rodríguez Fraile, Javier Rodríguez Hernández, María Ángeles Ruiz Rodríguez, and Mikaela Zubillaga Gómez

H. U. Sagunto. Valencia

Alberto Belda, Sergio Canales, Xavi Cortes, Iratxe Jimenez, Andrea Mendizabal, Marta Quilis, Enrique Rodilla, Alicia Roldan, Maria Carmen Saez, and Alberto Saura.
